# A new species of *Scolopocryptops* Newport: a troglobitic scolopocryptopine centipede from a remarkable siliciclastic area of eastern Brazil (Scolopendromorpha, Scolopocryptopidae, Scolopocryptopinae)

**DOI:** 10.3897/zookeys.487.9262

**Published:** 2015-03-16

**Authors:** Amazonas Chagas-Jr, Maria Elina Bichuette

**Affiliations:** 1 Departamento de Biologia e Zoologia, Instituto de Biociências, Universidade Federal de Mato Grosso. Avenida Fernando Correa da Costa, 2367, Boa Esperança, CEP-78060-900, Cuiabá, MT, Brasil; 2 Laboratório de Estudos Subterrâneos, Departamento de Ecologia e Biologia Evolutiva, Universidade Federal de São Carlos. Rodovia Washington Luis, Km 235, CEP-13565-905, São Carlos, SP, Brasil

**Keywords:** Chilopoda, Cave, Chapada Diamantina, eastern Brazil, Taxonomy

## Abstract

We describe *Scolopocryptops troglocaudatus* **sp. n.**, a new troglobitic scolopocryptopine centipede species. The species was found in a remarkable siliciclastic karst area of Eastern Brazil, in three caves of the Chapada da Diamantina, in the state of Bahia. *Scolopocryptops troglocaudatus* **sp. n.** is close to *Scolopocryptops miersii* Newport, 1845 and *Scolopocryptops ferrugineus macrodon* (Kraepelin, 1903) but differs from them by troglomorphic features, such as depigmentation, long appendages and a thin cuticle. This new species is the second troglobitic scolopocryptopine described and is the first discovered in Brazil.

## Introduction

*Scolopocryptops* are blind scolopendromorphs with 23 pairs of legs, and the prefemora of the ultimate legs with one dorsomedial and one ventral spinous process. There are currently 24 species and eight subspecies known from the Americas, Greater and Lesser Antilles, West Africa and along the Pacific Rim of Asia from Japan to Indonesia and the Fiji Islands (Chagas-Jr 2008, 2010).

In the Neotropics, the genus includes seven species: *Scolopocryptops miersii* Newport, 1845, *Scolopocryptops melanostoma* Newport, 1845, *Scolopocryptops ferrugineus* (Linnaues, 1762), *Scolopocryptops denticulatus* Bücherl, 1946, *Scolopocryptops guacharensis* Manfredi, 1957, *Scolopocryptops piauhyensis* Chagas-Jr, 2004 and *Scolopocryptops spiculifer* (Bücherl, 1949); three non-nominal subspecies: *Scolopocryptops ferrugineus inversus* (Chamberlin, 1921), *Scolopocryptops ferrugineus macrodon* (Kraepelin, 1903) and *Scolopocryptops ferrugineus riveti* (Brölemann, 1919); and three doubtful species: *Scolopocryptops aurantiaca* Gervais, 1847, *Scolopocryptops quadrisulcatus* Daday, 1891 and *Scolopocryptops viridis* Gervais, 1847.

True troglobitic species in the family Scolopocryptopidae have been described from the subfamilies Kethopiinae and Newportiinae: *Thalkethops grallatrix* Crabill, 1960 was found in the caves of New Mexico in the USA (Crabill 1960, Shelley 2002), Newportia (Newportia) troglobia Chagas-Jr & Shelley, 2003 was reported from a cave in Mexico (Chagas-Jr and Shelley 2003), Newportia (Newportia) stoevi Schileyko, 2013 from a cave in Puerto Rico (Schileyko 2013), and Newportia (Newportia) spelaea Ázara & Ferreira, 2014 and Newportia (Newportia) potiguar Ázara & Ferreira, 2014, both of which were reported from caves in northeastern Brazil.

Within the subfamily Scolopocryptopinae, two species of the genus *Scolopocryptops* have been recorded to be present in caves: *Scolopocryptops guacharensis*, from Cueva Del Guacharo, and *Scolopocryptops ferrugineus*, collected in three caves – Cueva Gruxent Graciliano, Cueva del Bunceo and Cueva Alfredo Jahn, all of which are in Venezuela (Manfredi 1957, Chagas-Jr 2003, 2008). *Scolopocryptops ferrugineus* is a widespread species that lives in a hypogean environment, but it could also be occasionally found in caves. Conversely, *Scolopocryptops guacharensis* seems to be a troglobitic species because it is restricted to a single cave and has peculiar features, such as some degree of depigmentation and a different length of the ultimate legs, which could be interpreted as troglomorphisms. Here, we describe a new species of *Scolopocryptops* from Brazil, which is the second troglobite in the subfamily.

## Material and methods

The type and additional material were first collected and examined under a stereomicroscope and then fixed in 70% alcohol. Photographs and length measurements were taken using a Leica Stereomicroscope (M205C). The scales are in metric units (millimeters, mm) and were made from photographs of specimens taken on a computer screen. The descriptive terminology follows that reported by Lewis et al. (2005) and Bonato et al. (2010).

The repository acronyms are as follows: MNRJ – Museu Nacional, Rio de Janeiro, Rio de Janeiro, Brazil; UFMT – Universidade Federal de Mato Grosso; UFSCar – Universidade Federal de São Carlos, São Carlos, Brazil.

### Study area

**Figure 1.**
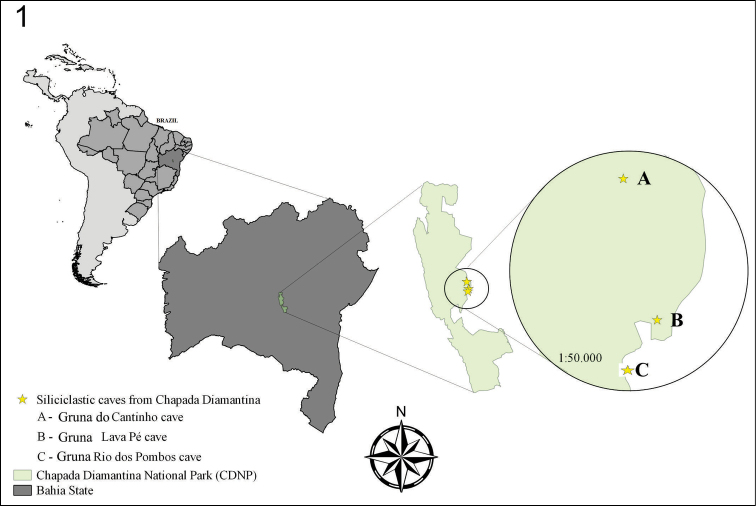
Map of the area where *Scolopocryptops troglocaudatus* sp. n. was found, Chapada Diamantina, Central Bahia. Author: D. M. von Schimonsky.

**Figure 2.**
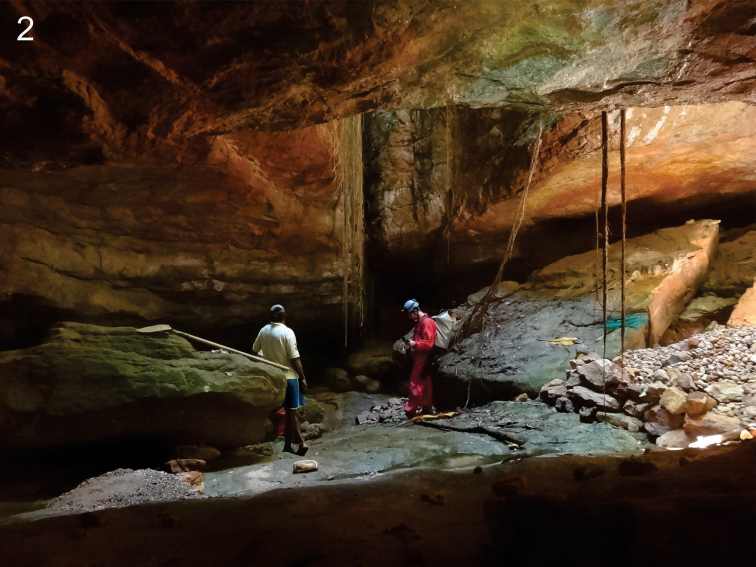
The entrance of Gruna do Cantinho Cave (type locality of *Scolopocryptops troglocaudatus* sp. n.) with roots and rock blocks. Author: E. C. Igual.

Chapada Diamantina is located in the central portion of the state of Bahia, eastern Brazil (Fig. 1). The caves where *Scolopocryptops troglocaudatus* sp. n. occurs were formed by siliciclastic (non-carbonatic sedimentary rocks) and quartzitic rocks inserted in a Mesoproterozoic Basin of the Chapada Diamantina Group, disposed in three layers, two of which formed from siliciclastic rocks (Schobbenhaus et al. 1984) (Fig. 2). Caves in this area are formed by the erosion of soft rock components by rainwater penetrating through surface cracks and leaving spaces delimited by the harder components. The caves are located in the Chapada Diamantina National Park (CDNP) and are thus under legal protection. However, most of the caves in the upper sector of Chapada Diamantina were heavily impacted by diamond mining in the past, an activity that extended to the early 1990s and continues to the present day (Bichuette et al. 2008).

## Taxonomy

### Order Scolopendromorpha Pocock, 1895 Family Scolopocryptopidae Pocock, 1896 Subfamily Scolopocryptopinae Pocock, 1896 Genus *Scolopocryptops* Newport, 1845

#### Scolopocryptops troglocaudatus sp. n.

Taxon classificationAnimaliaScolopendromorphaScolopocryptopidae

http://zoobank.org/F8B7359F-7FCD-4EC9-86C3-31F168F61919

Figs 3 , 4–7 , 8–9 , 10–13 , 14–16 , 17–19 , 20

Scolopocryptops sp. n. in Gallão and Bichuette (2015).

##### Type material examined.

Holotype unsexed (MNRJ) collected by Gallão, JE., Igual, EC. and von Schimonsky, DM. on 01.iv.2013 in Gruna do Cantinho Cave, Igatu, Andaraí, Bahia, Brazil.

##### Additional material examined.

Two juveniles (UFMT), two juveniles (UFSCar) all collected by Gallão, JE., Igual, EC. and von Schimonsky, DM. on 31.iii.2013 in Gruna Rio dos Pombos Cave, Igatu, Andaraí, Bahia, Brazil.

##### Etymology.

The name *troglocaudatus* is in allusion to the troglobitic status and the longest ultimate legs in the subfamily Scolopocryptopinae. This is from Latin *troglo*, meaning “cave”, and *caudatus*, meaning “with a tail”.

##### Diagnosis.

*Scolopocryptops* with a straight anterior margin of the forcipular coxosternum; tooth-plates formed by two long thickened chitinous layers, not fused with each other, more elevated on the sides than in the middle; without a pair of spiracles in the seventh pedal segment; ventral spinous process of the prefemur of the ultimate pair of legs short (small), and a very short dorsomedial spinous process; femur of the ultimate pair of legs longer than the prefemur and tibia.

##### Description of holotype.

*Length*: length of body (anterior margin of cephalic plate to posterior margin of tergite 23) 45 mm.

**Figure 3.**
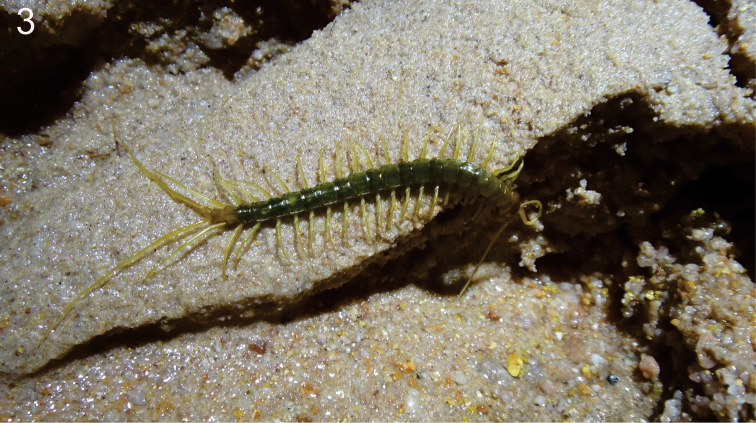
A live specimen showing the greenish coloration, displaying autogrooming in the natural habitat (Gruna do Cantinho Cave). Author: E. C. Igual.

*Pigmentation in life*: cephalic plate, first and last pedal segment, and coxosternite orange; body and pedal segments greenish, legs 1 to 21 and antennae light yellow, last two pairs of legs pale (Fig. 3). *Pigmentation in alcohol*: cephalic plate, coxosternite, tergites and sternites light brown and legs orange.

**Figure 4–7.**
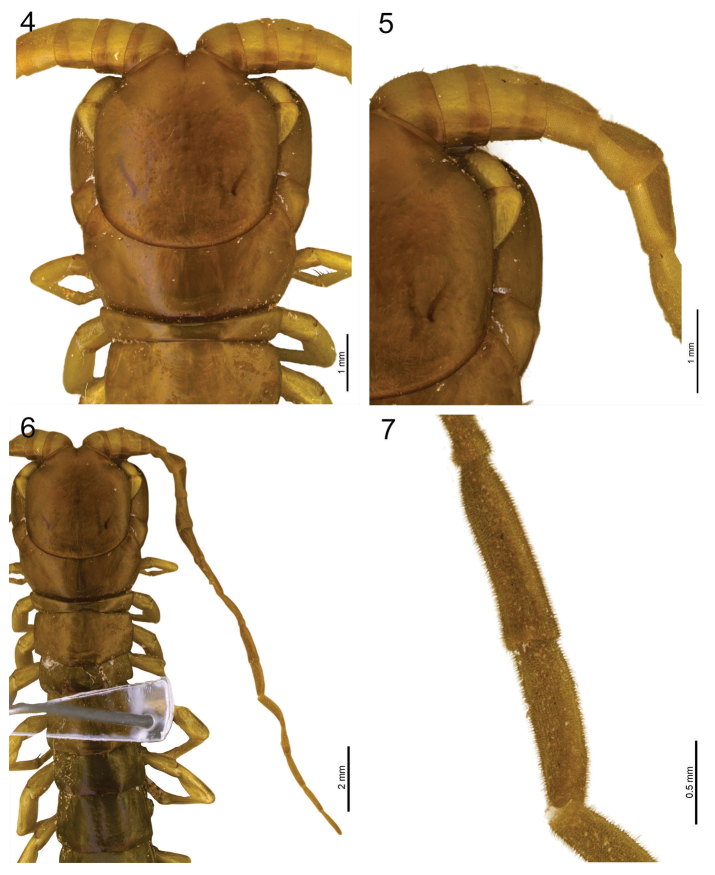
**4** *Scolopocryptops troglocaudatus* sp. n. Holotype (MNRJ). Cephalic plate and first two tergites. **5** *Scolopocryptops troglocaudatus* sp. n. Holotype (MNRJ). Part of the cephalic plate showing the first two basal articles of the right antenna **6** *Scolopocryptops troglocaudatus* sp. n. Holotype (MNRJ). Cephalic plate and the first eight tergites showing the length of the right antenna **7** *Scolopocryptops troglocaudatus* sp. n. Holotype (MNRJ). Eleventh and twelfth articles showing the length and width. Scale bar for Figure 4, 5 = 1 mm; 6 = 2 mm; 7 = 0.5 mm.

*Cephalic plate*: slightly longer than wider (length: 3.4 mm; width: 3.2 mm), smooth, without marginal ridges, sutures, sulci or depressions, its posterior margin overlying tergite 1 (Fig. 4).

*Antennae*: extending to the posterior border of T10 (Fig. 5); 17 articles; the first two basal articles and dorsal 1/3 of the third glabrous (Fig. 6); from the third to twelfth or seventeenth with short bristles covering all articles; first three basal articles wider than longer (length: 0.6 mm; width: 0.9 mm); fourth to seventeenth articles longer than wider; distal articles three times longer than wider (length: 1.1 mm; width: 0.3 mm) (Fig. 7).

**Figure 8–9.**
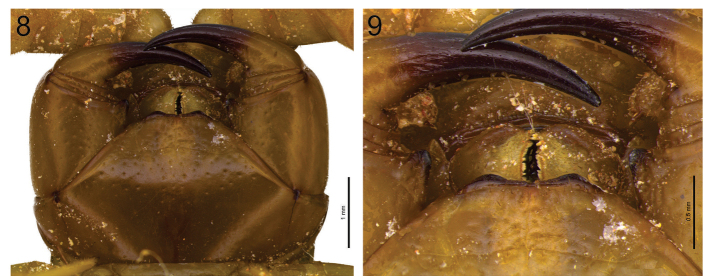
**8** *Scolopocryptops troglocaudatus* sp. n. Holotype (MNRJ). Forcipular coxosternum **9** *Scolopocryptops troglocaudatus* sp. n. Holotype (MNRJ). Tooth plates. Scale bar for Figure 8 = 1 mm; 9 = 0.5 mm.

*Forcipular coxosternum*: anterior margin straight, with a longitudinal suture ending in the middle of the transversal suture (Fig. 8); tooth-plates formed by two long thickened chitinous layers, not fused with each other, more elevated on the sides than in the middle. Process of forcipular trochanteroprefemur short, apex truncated (Fig. 9).

**Figure 10–13.**
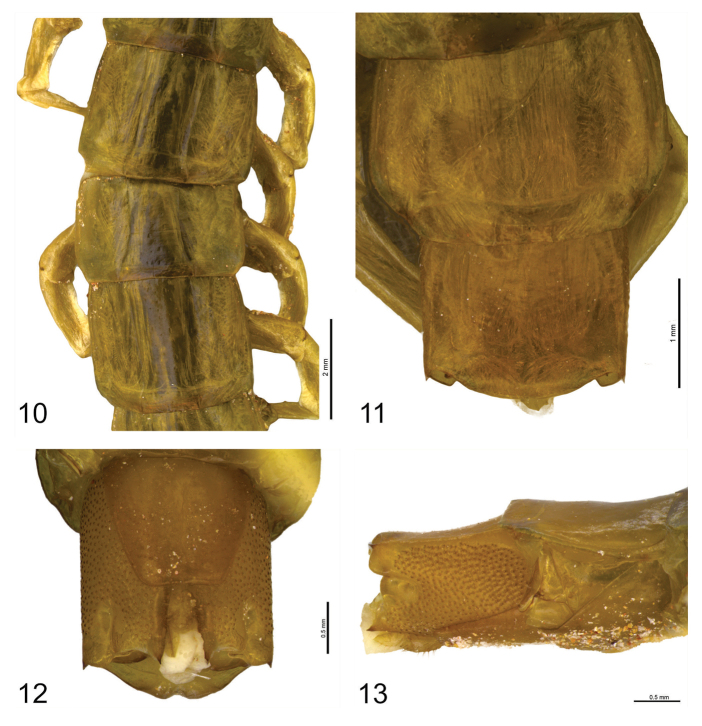
**10** *Scolopocryptops troglocaudatus* sp. n. Holotype (MNRJ). Tergites 12, 13 and 14 **11** *Scolopocryptops troglocaudatus* sp. n. Holotype (MNRJ). Tergites 22 and 23 **12** *Scolopocryptops troglocaudatus* sp. n. Holotype (MNRJ). Segment 23. Ventral view **13** *Scolopocryptops troglocaudatus* sp. n. Holotype (MNRJ). Segment 23. Lateral view. Scale bar for Figure 10 = 2 mm; 11 = 1 mm; 12, 13 = 0.5 mm.

*Tergites*: smooth, with very light fine punctuation. Tergite 1 with an anterior transversal sulcus, but without sutures; T3 to T7 with incomplete short paramedian sutures; T8 to T20 with complete paramedian sutures (Fig. 10). Tergites 6 (or 7) to T22 with margination. Tergite of ultimate leg-bearing segment without margination, but with a membranous line separating tergite and coxopleura (Fig. 11); posterior border with a low longitudinal depression and a convex posterior margin.

*Spiracles*: not present in the seventh pedal segment.

*Sternites*: smooth, wider than longer. Sternite of ultimate leg-bearing segment narrow posteriorly, longer than wide, posterior margin straight (Fig. 12).

*Coxopleuron*: Coxopleural process short, parallel and pointed. Pore field reaching almost the whole area of the coxopleura, except the dorsal and posterior areas and the medial depression, its posterior corner ending at a strong, sclerotized point (Fig. 13).

*Legs*: Legs 1 to 21 with undivided tarsus, legs 22 and 23 with tarsi 1 and 2. Legs 1 to 19 with two tibial spurs, legs 20 and 21 with one lateral tibial spur, legs 22 and 23 without spurs; legs 1 to 21 with one lateral tarsal spur, legs 22 and 23 without. Pretarsus of legs 1 to 21 with well-developed pairs of accessory spurs, accessory spurs on legs 22 and 23 very short.

**Figure 14–16.**
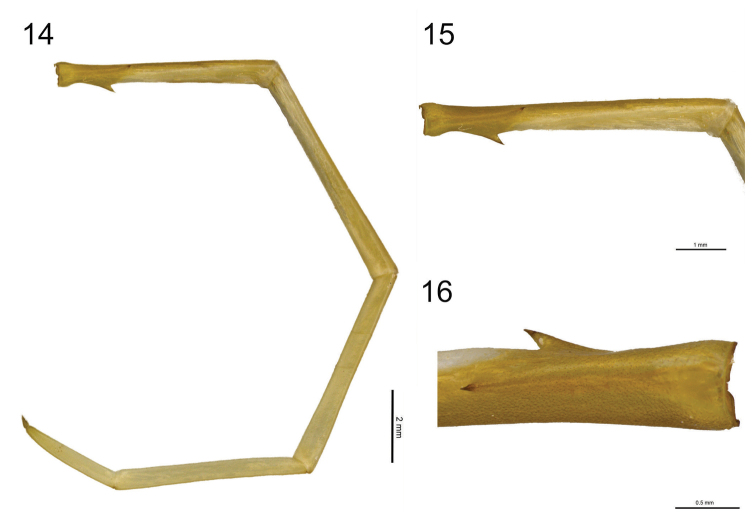
**14** *Scolopocryptops troglocaudatus* sp. n. Holotype (MNRJ). Ultimate right leg **15** *Scolopocryptops troglocaudatus* sp. n. Holotype (MNRJ). Prefemur of the ultimate right leg **16** *Scolopocryptops troglocaudatus* sp. n. Holotype (MNRJ). Dorsomedial and ventral spinous processes of the prefemur of the ultimate leg. Scale bar for Figure 14 = 2 mm; 15 = 1 mm; 16 = 0.5 mm.

Ultimate pair of legs smooth, longer and slender (length: 26.2 mm) (Fig. 14). Ventral spinous process of the prefemur short (small) and the dorsomedial spinous process very short (Figs 15 and 16). Femur longer (length: 6.1 mm) than the prefemur (length: 6.0 mm) and tibia (5.7 mm); tarsus 1 (length: 5.3 mm), tarsus 2 (length: 2.7 mm) and pretarsus (length: 0.4 mm).

##### Type locality.

Gruna do Cantinho Cave, Igatu, Andaraí, Bahia, Brazil.

##### Distribution.

Gruna do Cantinho Cave, Gruna Rio dos Pombos Cave and Gruna Lava Pé Cave, all Caves from Chapada Diamantina, central Bahia, Eastern Brazil (Fig. 1).

##### Remarks on juveniles.

**Figure 17–19.**
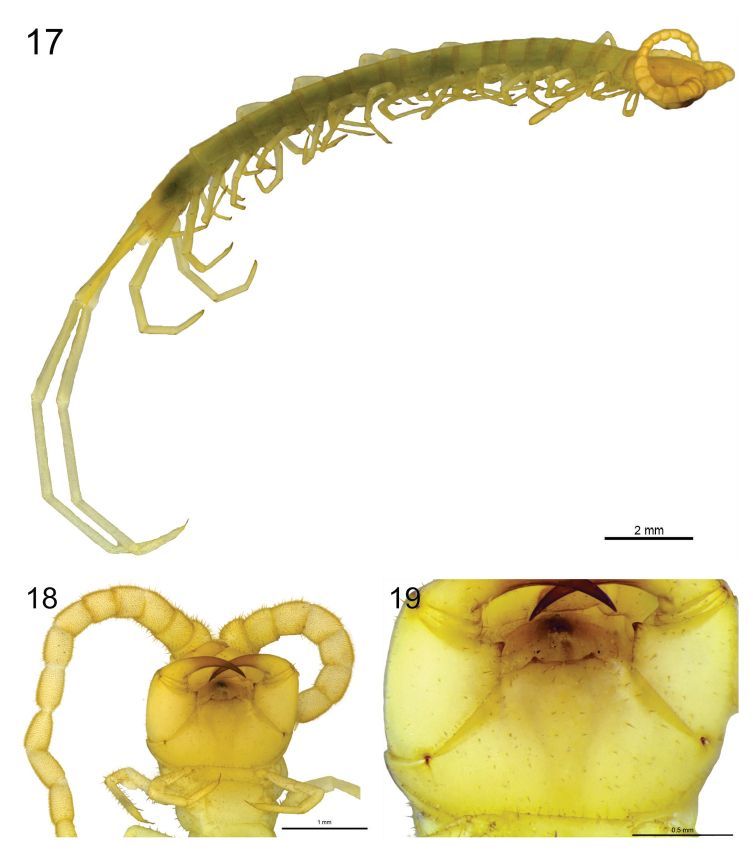
**17** *Scolopocryptops troglocaudatus* sp. n. Juvenile (UFSCar). Habitus **18** *Scolopocryptops troglocaudatus* sp. n. Juvenile (UFSCar). Forcipular coxosternum **19** *Scolopocryptops troglocaudatus* sp. n. Juvenile (UFSCar). Tooth plates. Scale bar for Figure 17 = 2 mm; 18 = 1 mm; 19 = 0.5 mm.

**Figure 20–21.**
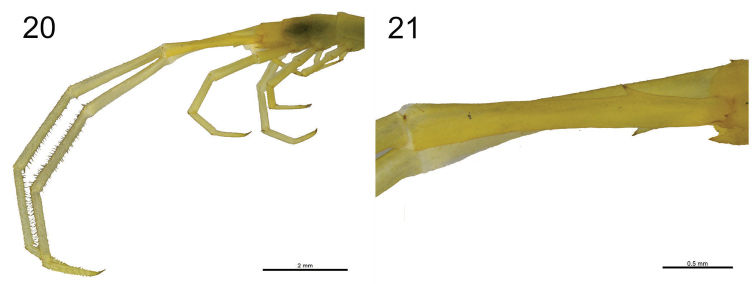
**20** *Scolopocryptops troglocaudatus* sp. n. Juvenile (UFSCar). Ultimate pair of legs showing the length of the articles and the pilosity of the tibiae and tarsi **21** *Scolopocryptops troglocaudatus* sp. n. Juvenile (UFSCar). Prefemur of the right ultimate leg showing the dorsomedial and ventral spinous processes. Scale bar for Figure 20 = 2 mm; 21 = 0.5 mm.

The color pattern of the juveniles: antennae, cephalic plate, first and last pedal segments, and coxosternite light orange (Fig. 17); from T2 to T22 light greenish; legs pale. The first two basal articles of the antennae pilose, with long and short setae. The integument of the cephalic plate, coxosternite (Fig. 18), tergites, sternites and legs pilose, with long and fine setae. Prefemora and femora of ultimate legs with few long and short setae. The tooth-plates are formed by two long thickened chitinous layers, not fused with each other, more elevated on the sides than in the middle (Fig. 19). The margins of the sides of the tooth plates are pointed. Process of the forcipular trochanteroprefemur short, and apex pointed. Coxopleural processes median in length, parallel and pointed. Ventral and dorsalmedial spinous processes of the prefemur of the ultimate legs longer than holotype (Figs 20 and 21).

##### Habitat and habits.

*Scolopocryptops troglocaudatus* sp. n. adults were observed exposed in the substrate formed by humid sand (Fig. 2). The adults are apparently indifferent to light, showing a very calm behavior when lit in the natural habitat. In contrast, the juveniles were collected buried in the humid sand, which represented a more cryptobiotic habit.

## Discussion

So far, at least 18 species of Scolopendromorpha have been described from caves worldwide, and five of these species are found in Brazil. Considering the Scolopocryptopinae, the species described herein represents the second troglobitic (restricted to subterranean environments) species in the world. The first troglobitic scolopocryptopine was described after its discovery in a cave in Venezuela by Manfredi (1957) as *Otocryptops ferrugineus guacharensis* Manfredi, 1957. Chagas-Jr (2003) revised the taxonomic status of the species and compared it with *Scolopocryptops ferrugineus*. He concluded that *Otocryptops ferrugineus guacharensis* was exclusive to the Cueva del Guacharo and showed that some troglomorphic features, such as depigmentation and long legs, were strong evidence that *Scolopocryptops guacharensis* was restricted to subterranean life (Chagas-Jr 2003). *Scolopocryptops troglocaudatus* sp. n. shares some troglomorphic characters with *Scolopocryptops guacharensis* such as depigmentation, the length of the antennae, the length of the ultimate pair of legs, and the pilosity of the tibia and tarsi of ultimate pair of legs.

*Scolopocryptops troglocaudatus* sp. n. resembles *Scolopocryptops miersii* in having a straight anterior margin of the forcipular coxosternum and tooth-plates formed by two long thickened chitinous layers, which are not fused with each other and are more elevated on the sides than in the middle. However, *Scolopocryptops troglocaudatus* sp. n. differs from *Scolopocryptops miersii* in the length of the ultimate pair of legs, the length of the coxopleural process, and the length of the dorsomedial and ventral spinous process of the prefemur of the ultimate pair of legs. In addition, there is no pair of spiracles on the seventh pedal segment of *Scolopocryptops troglocaudatus* sp. n.

A noteworthy characteristic is the length of the ultimate pair of legs in *Scolopocryptops troglocaudatus* sp. n., which is almost the half of the length of the body, whereas their length in *Scolopocryptops miersii* is short, never reaching even half the length of the body. The dorsomedial and ventral spinous process in the prefemur of the ultimate legs in *Scolopocryptops troglocaudatus* sp. n. are short and small, whereas those in *Scolopocryptops miersii* are long and large.

*Scolopocryptops troglocaudatus* also resembles *Scolopocryptops ferrugineus macrodon* in the length of the coxopleural process, which is short in both taxa, but differs from *Scolopocryptops ferrugineus macrodon* in the anterior margin of the forcipular coxosternum, the shape of the tooth-plates, the length of the ultimate pair of legs, and the length of the dorsomedial and ventral spinous processes of the prefemur of the ultimate pair of legs. The anterior margin of *Scolopocryptops ferrugineus macrodon* is almost straight; the tooth-plates are formed by two chitinous lobes, sometimes with a chitinous crest, with its margin being slightly granulated. The length of the ultimate pair of legs and the length of the dorsomedial and ventral spinous processes of the prefemur of the ultimate pair of legs are very similar to *Scolopocryptops miersii* but very different from that described for *Scolopocryptops troglocaudatus* sp. n.

**Endemism.** *Scolopocryptops troglocaudatus* sp. n. is most likely endemic existing only in siliciclastic caves from Igatu, occurring in an area of approximately 10 km^2^. This statement is corroborated by the numerous collections conducted by the Laboratório de Estudos Subterrâneos team in the region since 2006, when no specimens were found in the limestone caves close to these. Other troglobitic and endemic species occur in this region, and the area is clearly an area of high diversity for terrestrial cave invertebrates, with at least 20 unique troglobitic invertebrates distributed in a 25 km^2^ area (Gallão and Bichuette 2015).

**Troglomorphic traits and troglobitic status.** Troglomorphic organisms in general are highly homoplastic, widely known for reduced eyes and melanic pigmentation, a phenomenon also observed to be related to the behavioral traits (Trajano and Bockmann 1999, Parzefall and Trajano 2010). These characters are not necessarily adaptive, unless pleiotropic effects have been shown (Jeffery 2010). The lack of eyes is shared by all species of Scolopocryptopidae and depigmentation and is shared by many other characters suggesting troglomorphisms must be verified because these are not sufficient to prove the cave-restricted status for a scolopocryptopid species. For Scolopocryptopinae, we suggest that the last two pairs of legs represent a possible troglomorphism because they are very distinct in length compared with other species. The adult *Scolopocryptops troglocaudatus* sp. n. shows greenish body coloration, with pale yellow legs and head. On the contrary, the juvenile individuals show a pale aspect in the entire body, including the appendages. These differences in the pigmentation must be reported in several studies that try to detect troglomorphisms, or equivocal classifications can be proposed. Even with the absence of one typical troglomorphism (reduction of melanic pigmentation), the non-occurrence of the species outside the caves clearly indicates the troglobitic status of *Scolopocryptops troglocaudatus* sp. n.

Considering other character-states, we detected at least three troglomorphisms in *Scolopocryptops troglocaudatus* sp. n.: an extremely long ultimate pair of legs (exceeding 2/3 of the body length: 26.2 mm), a long antennae and a reduced sclerotization of the cuticle. The long antennae and the reduced cuticle are most likely related to optimization of the detection of chemical and mechanical stimuli and to the intolerance for desiccation. Caves are extremely humid habitats, and troglobitic arthropods show, in general, a reduction in the cuticle (Barr 1968). There is no information about the function of the last pair of legs in the subfamily Scolopocryptopinae, and it is not possible to draw any conclusions about its importance with regard to the isolation in subterranean habitats, the detection of prey and/or even defense of territory.

**Conservation remarks.** Caves are unique habitats that are often inhabited by relict taxa showing high degree of endemism (Trajano and Bichuette 2010). The region of Igatu, Chapada Diamantina shows a high diversity of troglobites and some relict taxa, being the type-locality of four troglobites: the harvestman *Discocyrtus pedrosoi* Kury, 2008, the catfish *Glaphyropoma spinosum* Bichuette, Pinna & Trajano, 2008, the scorpion *Troglorhopalurus translucidus* Lourenço, Baptista & Giupponi, 2004 and the mygalomorph spider *Tmesiphantes hypogeus* Bertani, Bichuette & Pedroso, 2013. Furthermore, the region shows endemisms and phylogenetical and geographical relicts as, for example, the collembolan of *Verhoefiella* genus, previously only thought to have Palaeartic distribution (Gallão and Bichuette 2015).

The new species described herein represents the sixth troglobite described for the region, and its restricted distribution (three caves in a 10 km^2^ area) categorizes it as fragile in terms of conservation criteria.

## Supplementary Material

XML Treatment for Scolopocryptops troglocaudatus
